# Graphitic Carbon Nitride as a New Sustainable Photocatalyst for Textile Functionalization

**DOI:** 10.3390/polym13152568

**Published:** 2021-07-31

**Authors:** Jelena Vasiljević, Ivan Jerman, Barbara Simončič

**Affiliations:** 1 Faculty of Natural Sciences and Engineering, University of Ljubljana, Aškerčeva 12, 1000 Ljubljana, Slovenia; jelena.vasiljevic@ntf.uni-lj.si; 2 National Institute of Chemistry, Hajdrihova 19, 1000 Ljubljana, Slovenia; ivan.jerman@ki.si

**Keywords:** graphitic carbon nitride, textile, fibres, functionalization, surface and bulk modification, photocatalytic activity, self-cleaning, antibacterial properties

## Abstract

As a promising organic semiconducting material, polymeric graphitic carbon nitride (g-C_3_N_4_) has attracted much attention due to its excellent optical and photoelectrochemical properties, thermal stability, chemical inertness, nontoxicity, abundance, and low cost. Its advantageous visible light-induced photocatalytic activity has already been beneficially used in the fields of environmental remediation, biological applications, healthcare, energy conversion and storage, and fuel production. Despite the recognized potential of g-C_3_N_4_, there is still a knowledge gap in the application of g-C_3_N_4_ in the field of textiles, with no published reviews on the g-C_3_N_4_-functionalization of textile materials. Therefore, this review article aims to provide a critical overview of recent advances in the surface and bulk modification of textile fibres by g-C_3_N_4_ and its composites to tailor photocatalytic self-cleaning, antibacterial, and flame retardant properties as well as to create a textile catalytic platform for water disinfection, the removal of various organic pollutants from water, and selective organic transformations. This paper highlights the possibilities of producing g-C_3_N_4_-functionalized textile substrates and suggests some future prospects for this research area.

## 1. Introduction

**Figure 1:**
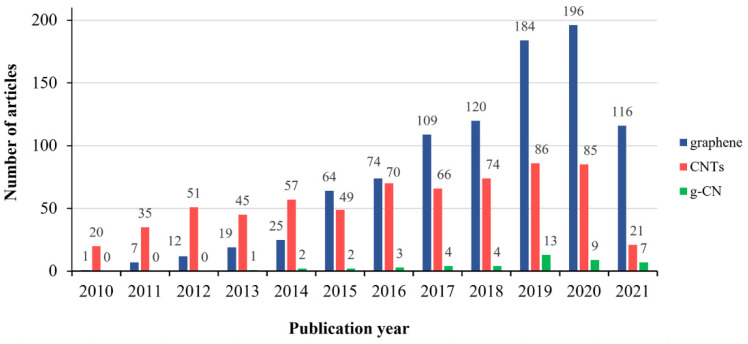
The number of publications related to graphene, CNTs, and g-C_3_N_4_ in textiles since 2010, retrieved using the keywords “graphene or reduced graphene oxide or rGO or r-GO” (“CNTs or carbon nanotubes,” “graphitic carbon nitride or carbon nitride or g-C_3_N_4_ or g-CN”) in the title and the words “textile of fabric or fabrics” in the abstract (Source: Web of Science (https://www.webofscience.com/wos/woscc/basic-search), accessed on 25 July 2021).

Increased awareness of the importance of sustainable development and environmentally friendly approaches in various technological fields, including chemical textile finishing, has dictated the introduction of “green” finishing agents and non-hazardous protocols for the chemical modification of textile substrates. Among these “green” alternatives, carbon-based materials, such as graphene-based nanomaterials and carbon nanotubes (CNTs), have already been advantageously used for the functionalization of textile surfaces due to their superior chemical, physical, thermal, and electrical properties as well as their biocompatibility and low toxicity (Figure 1). These carbon-based materials have already established themselves as effective photocatalysts alongside titanium dioxide, which remains the most attractive material for textile functionalization [1,2,3]. In contrast to graphene and carbon nanotubes, graphitic carbon nitride (g-C_3_N_4_), which also belongs to the group of carbon-based nanomaterials, seems to be virtually unexplored in textile applications (Figure 1).

**Figure 2:**
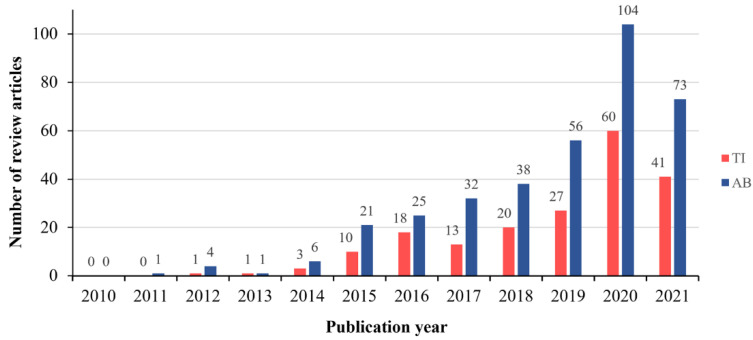
The number of review articles related to g-CN containing the words “graphitic carbon nitride or g-C_3_N_4_ or g-CN” in the title and the word “review” in the abstract (TI) or the words “graphitic carbon nitride or g-C_3_N_4_ or g-CN and review” in the abstract (AB) (Source: Web of Science (https://www.webofscience.com/wos/woscc/basic-search), accessed on 25 July 2021).

In the last decade, g-C_3_N_4_, a next-generation sustainable metal-free polymeric n-type semiconductor photocatalyst, has attracted much attention due to its excellent optical and photoelectrochemical properties, thermal stability, chemical inertness, nontoxicity, abundance, and low cost [4]. It has emerged as a promising organic semiconducting material that could efficiently replace inorganic semiconductors in environmental remediation, energy conversion/storage, fuel production, and healthcare. As shown in Figure 2, there were more than 300 review articles on g-C_3_N_4_ from 2010 to 2021, and the number of publications is rapidly increasing every year.

**Figure 3:**
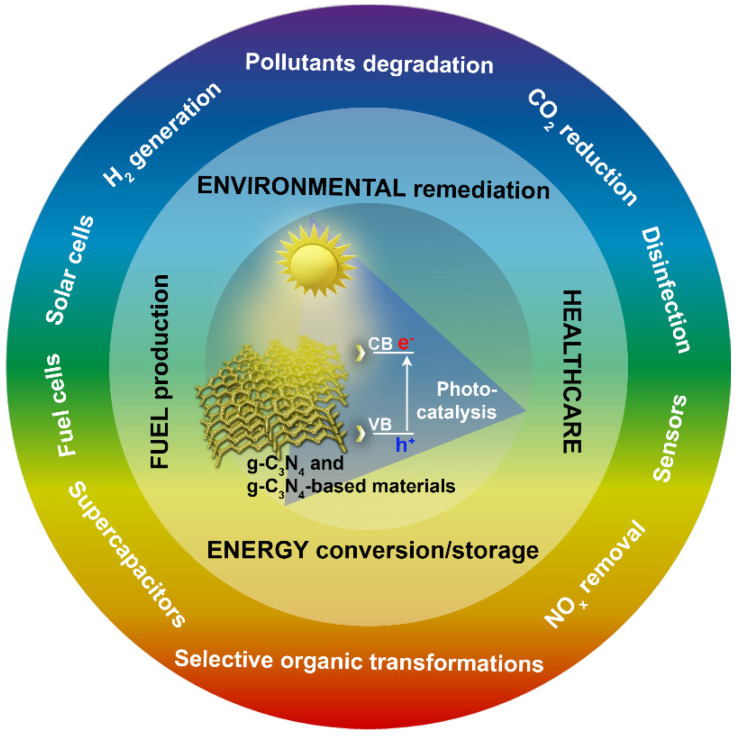
Schematic presentation of the fundamentals of the photocatalysis of g-C_3_N_4_- and g-C_3_N_4_-based materials and their potential applications in the fields of environmental remediation, healthcare, energy conversion and storage, and fuel production.

These review articles were mainly concerned with the synthesis and design of g-C_3_N_4_ and g-C_3_N_4_-based nanomaterials, their photocatalytic mechanisms, structural modifications, and the development of energy and environmental applications, such as the effective conversion of solar energy to generate H_2_ through water splitting; energy conversion and storage; sensors; solar cells; fuel cells; supercapacitors; the photoreduction of CO_2_; the removal of NO_x_; selective organic transformations; the photocatalytic degradation of various pollutants from wastewater, soil, and air; and bacterial disinfection (Figure 3) [5,6,7,8,9,10,11,12,13,14,15,16,17,18,19]. Design strategies for biological applications, biosensors, and electrochemical devices were also discussed in detail [15,16,17,20,21].

However, to the best of our knowledge, there is still no published review on the functionalization of textiles with g-C_3_N_4_- and g-C_3_N_4_-based materials. This represents an important knowledge gap regarding g-C_3_N_4_ as an emerging “green” nanomaterial for the functionalization of textiles. Therefore, we aim to provide a critical review of recent advances in the surface and bulk chemical modification of textile fibres by g-C_3_N_4_ and g-C_3_N_4_ nanocomposites with other semiconductors or in combination with conventional finishing agents to provide photocatalytic self-cleaning, antibacterial, and flame-retardant properties as well as a textile catalytic platform for water disinfection and the removal of various organic pollutants from water.

## 2. Properties of g-C_3_N_4_

**Figure 4:**
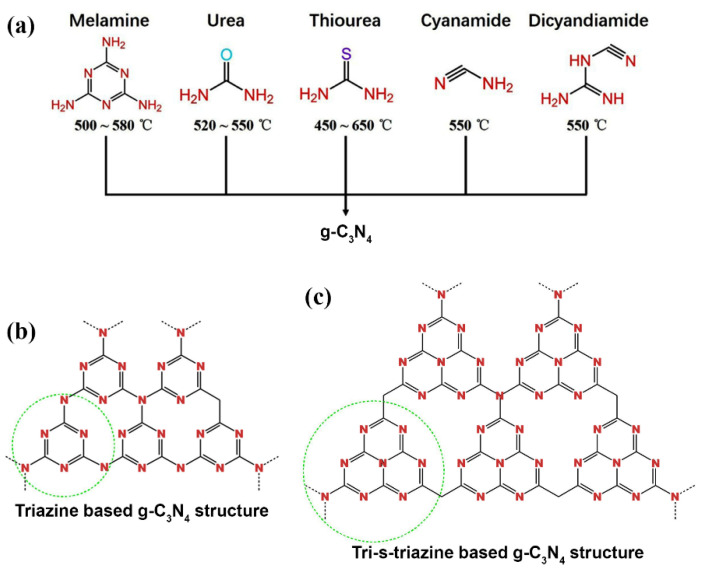
(**a**) Molecular structures of the g-C_3_N_4_ precursors and the corresponding temperatures used for the thermal polycondensation process. Reprinted with permission from [31], Copyright 2021, Elsevier. (**b**,**c**) Molecular structures of triazine (**b**) and tri-s-triazine (**c**) based on potential g-C_3_N_4_ allotropes. Reprinted with permission from [32], Copyright 2020, Elsevier.

**Figure 5:**
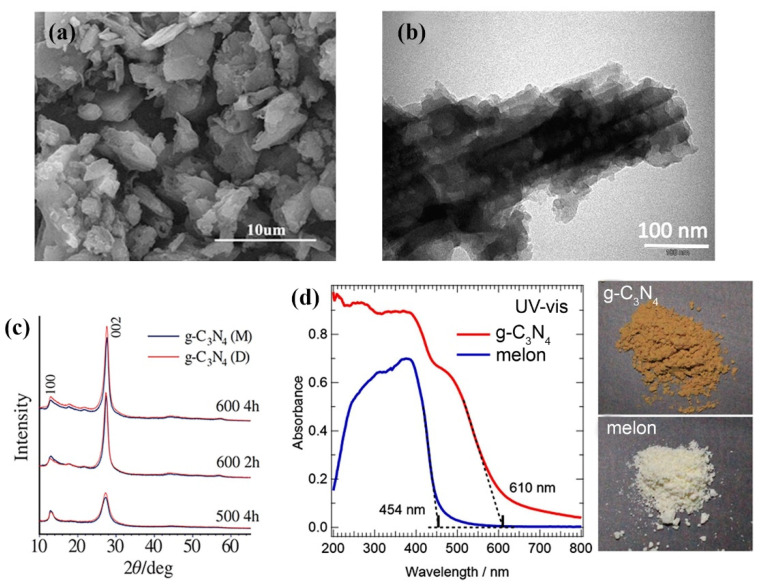
(**a**) SEM image of g-C_3_N_4_ prepared from melamine. Reprinted with permission from [26], Copyright 2019, Elsevier. (**b**) TEM image of g-C_3_N_4_ prepared from melamine. Reprinted with permission from [27], Copyright 2020, Elsevier. (**c**) XRD patterns of g-C_3_N_4_ (M) and g-C_3_N_4_ (D) prepared from melamine and dicyandiamide, respectively. Reprinted with permission from [28], Copyright 2021, Elsevier. (**d**) UV-vis spectra of the g-C_3_N_4_ and melon with the corresponding photographs of the powdery samples used for this measurement. Reprinted with permission from [29], Copyright 2020, Elsevier.

g-C_3_N_4_ is usually prepared through the thermal polymerization of nitrogen-rich precursors such as melamine, urea, thiourea, dicyandiamide, and cyanamide (Figure 4a) [22]. This thermal polymerization process, which occurs in the temperature range of 450–650 °C, allows for the subsequent conversion of these precursors to melam, melem, and melon, which subsequently, via polycondensation polymerization reactions, transform into a two-dimensional layered structure of sp^2^ hybridized C and N atoms, i.e., g-C_3_N_4_. The resulting g-C_3_N_4_ is not soluble in acidic, alkaline, or organic solvents [23,24]. The g-C_3_N_4_ layers are mainly bound by van der Waals forces and consist of s-triazine (C_3_N_4_) rings and tri-s-triazine (C_6_N_7_) rings (Figure 4b,c). The final morphology, pore size, band gap performance, and photocatalytic properties are strictly dependent on the precursor type and the production parameters [17,20,21,25].

g-C3N4 is characterized by a two-dimensional lamellar structure [26,27], as shown in the representative SEM and TEM images of the g-C3N4 prepared from melamine in Figure 5a,b. The lamellar structures loosely agglomerate between each other, resulting in microsized g-C3N4 particles of irregular shapes. The XRD pattern of the g-C3N4 is characterized by the dominant diffraction peak located at 2θ~27.5° and the second peak of lower intensity located at 2θ~13.1° [28]. The representative XRD patterns of the g-C3N4 produced from melamine (g-C3N4 (M)) and dicyandiamide (g-C3N4 (D)) are shown in Figure 5c. The dominant diffraction peak can be assigned to the (002) plane and characterizes the distance between the parallel conjugated aromatic C-N 2D layers, whereas the second diffraction peak can be attributed to the (100) diffraction plane and characterizes the distance between the tri-s-triazine units within the 2D C-N layer.

The colour of g-C_3_N_4_ ranges from light yellow to brownish [29,30]. The photographs of the powdered form of melon and g-C_3_N_4_ obtained from melamine are presented in Figure 5d, which also shows the corresponding UV–vis absorption spectra. The significant redshift of the absorption edge from 454 nm for melon to 610 nm for g-C_3_N_4_ indicates the lowering of an energy gap with the thermal polymerization.

The moderate energy of the band gap of g-C_3_N_4_ (*E*_g_ ≈ 2.7 eV) enables a visible-light- driven (*λ* ≈ 450–460 nm) photocatalytic reaction, while the efficiency of the photocatalytic activity is affected by the adsorption capacity of the g-C_3_N_4_ material [17]. The adsorption capacity is greatly enhanced by both the electron-rich nitrogen functional groups and existing defects on the g-C_3_N_4_ surface or their oxidation [20]. The increase in the number of photocatalytically active g-C_3_N_4_ sites is mainly enhanced by the inhibition of the *π*–*π* interaction between the stacking layers, i.e., by modifying the chemical structure of the precursor or by exfoliation processes [33,34].

**Figure 6:**
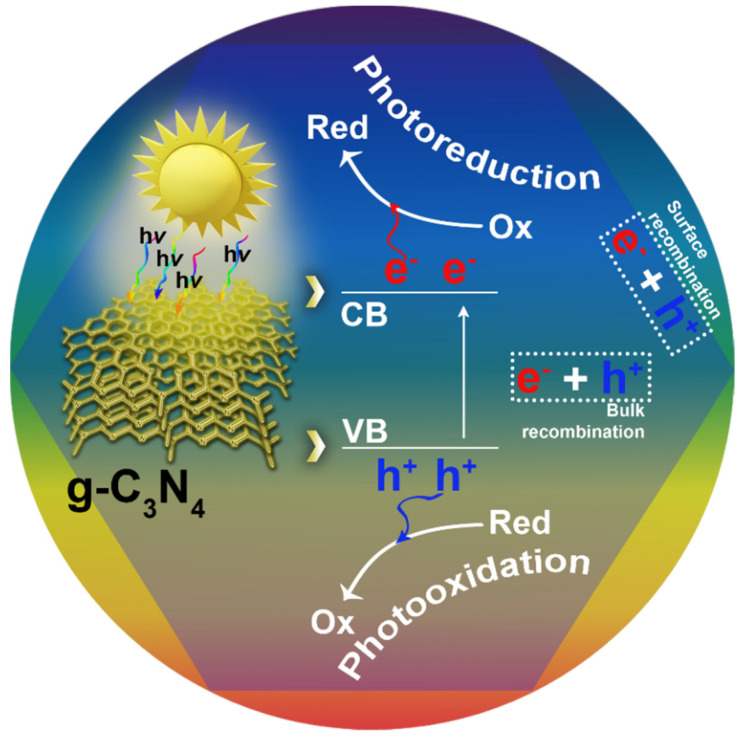
Schematic illustration of the photoexcitation process in g-C_3_N_4_ and the formation of electron–hole pairs involving redox reactions and/or bulk/surface recombination processes.

As a photocatalyst, g-C_3_N_4_ acts as an n-type semiconductor; the absorption of the photons with energies greater than the band gap energy leads to the transfer of electrons from the valence band to the conduction band, resulting in the formation of electron–hole (e^−^–h^+^) pairs (Figure 6). Therefore, the valence band acquires oxidation power, while the conduction band acquires reduction power. Afterwards, the photoproduced e^−^–h^+^ pairs can participate in redox reactions with electron donor/acceptor species adsorbed on the surface of the g-C_3_N_4_ materials [17]. The other possibility for the photoproduced e^−^–h^+^ pairs is the transition of electrons from the conduction band back to the valence band, which is an undesirable recombination process followed by energy loss (heat release or light emission). In agreement with the oxidation/reduction potentials of the g-C_3_N_4_ h^+^ and e^−^ [35], the photogenerated e^−^ reduces absorbed O_2_ to O_2_^∙^^−^ radicals, which, together with h^+^, participate in the decomposition process of organic pollutants to CO_2_ and H_2_O [17]. The photogenerated electrons are capable of reducing CO_2_ and H_2_O, which is the reason for the wide application of the g-C_3_N_4_ material for photocatalytic CO_2_ reduction, water splitting, and disinfection [21]. The moderate oxidation ability of the photogenerated h^+^ of the g-C_3_N_4_ prevents the production of the unselective strong ^∙^OH radicals, which enables the use of the g-C_3_N_4_ material for the selective photooxidation processes with the excluded production of the CO_2_ by ^∙^OH radicals [17,21].

Various modification strategies have been employed in g-C_3_N_4_ materials to improve the photocatalytic efficiency, which is inhibited by the high rate of e^−^–h^+^ recombination process and low charge mobility, the inability to absorb visible light below 460 nm, low surface area, and limited interfacial interactions [36]. Some of these strategies include heterojunction fabrication [17,22,37,38], metal doping [19], non-metal doping [39], and structural defect engineering [22,34].

## 3. Chemical Modification of Textiles by g-C_3_N_4_

To date, g-C_3_N_4_ has been applied to cotton [40,41,42,43,44,45,46], polyacrylonitrile [47,48,49,50], polyester and polyester/viscose fibres [51,52,53,54], polyethylene terephthalate [55,56], and polylactic acid [57]. Information about g-C_3_N_4_ precursors, g-C_3_N_4_ modification, the application method, and the performance of the textile surface functionalized by g-C_3_N_4_ is summarised in Table 1.

Prior to textile application, the ex-situ synthesis of g-C_3_N_4_ was conducted by thermal condensation of dicyandiamide, melamine, or urea precursors heated to 450 °C, 500 °C, or 550 °C and calcined from 2 to 5 h. Subsequently, the synthesised g-C_3_N_4_ was cooled to room temperature and ground into a powder. To prepare g-C_3_N_4_ nanosheets, exfoliation was conducted in water [46,53], H_2_SO_4_ [42] or in NaOH [44] under ultrasonic treatment. In addition to the unmodified material, the oxidised form of g-C_3_N_4_ [41], carboxyl-modified and carboxyl-3-triethoxysilylpropylamine-modified g-C_3_N_4_ [45] have also been used for textiles. Despite its many advantages, the in situ synthesis of g-C_3_N_4_ nanosheets in the presence of textile substrates is not possible due to the high temperatures required for g-C_3_N_4_ synthesis, which are not tolerated by the textile fibres and would lead to the textile’s thermal degradation.

To fabricate photocatalytic textiles, g-C_3_N_4_ was applied alone or in combination with other metal oxide-based semiconductors, such as TiO_2_ and Cu oxides/hydroxides [41,42,45,53,58] and the carbon-based semiconductor GO [40,58] with the aim of increasing the photocatalytic efficiency of the heterojunctions. Moreover, phytic acid was used in combination with g-C_3_N_4_ to provide additional functional properties such as flame retardancy [46].

To produce functional properties, both the surface and bulk chemical modifications of the textile substrates were conducted. For the surface modification, g-C_3_N_4_ was applied using the conventional pad–dry–cure technique [53], double-dip-double-nip padding, or immersion in the g-C_3_N_4_ suspension under suitable conditions followed by squeezing and drying with or without curing [40,41,43,45,51,54], spraying [44] as well as a layer-by-layer (LbL) self-assembly technique [42,46]. Electrospinning was used for fibre bulk modification [47,48,49,50,55,56,57].

To improve the adsorption ability and the adhesion of g-C_3_N_4_ to the fibre surface, electrostatic assembly techniques were used. One of these application techniques was the pretreatment of cotton fibres with poly(diallyldimethylammonium chloride) (PDDA) as a strong cationic polyelectrolyte, which provides electrostatically attractive interactions with the negatively charged g-C_3_N_4_ nanosheets, which are subsequently deposited on the PDDA-modified cotton [43,55,56]. Another was the LbL self-assembly technique, which involves a cyclic assembly of alternating deposition of the anionic g-C_3_N_4_ solution and the cationic TiO_2_ or polyethyleneimine solutions onto cotton fabrics with intervening rinsing and drying to form a multilayer structure of coatings [42,46]. In the case of synthetic fibres, low melting point sheath core composite polyester (LMPET) fibres were used to surface immobilize g-C_3_N_4_ during the curing (baking) step at temperatures higher than the melting temperature of the sheath layer [51,52,54].

Among the prominent textile functional properties produced by g-C_3_N_4_ are the photocatalytic self-cleaning of various stains [43,44]; the degradation of dyes [40,42,43,44,50]; water disinfection and antibacterial properties [40,45,47]; the degradation of antibiotics, bactericides, and insecticides [42,51,53,54,55,56,57]; the purification of oilfield produced water [49]; the sensing and photocatalytic detoxification of air pollutants [41,42,44]; and thermal stability and flame retardancy [46]. In addition to textile functionalities, the recyclability and stability of the g-C_3_N_4_ modified textile substrates have also been investigated [43,47,51,53]

**Figure 7:**
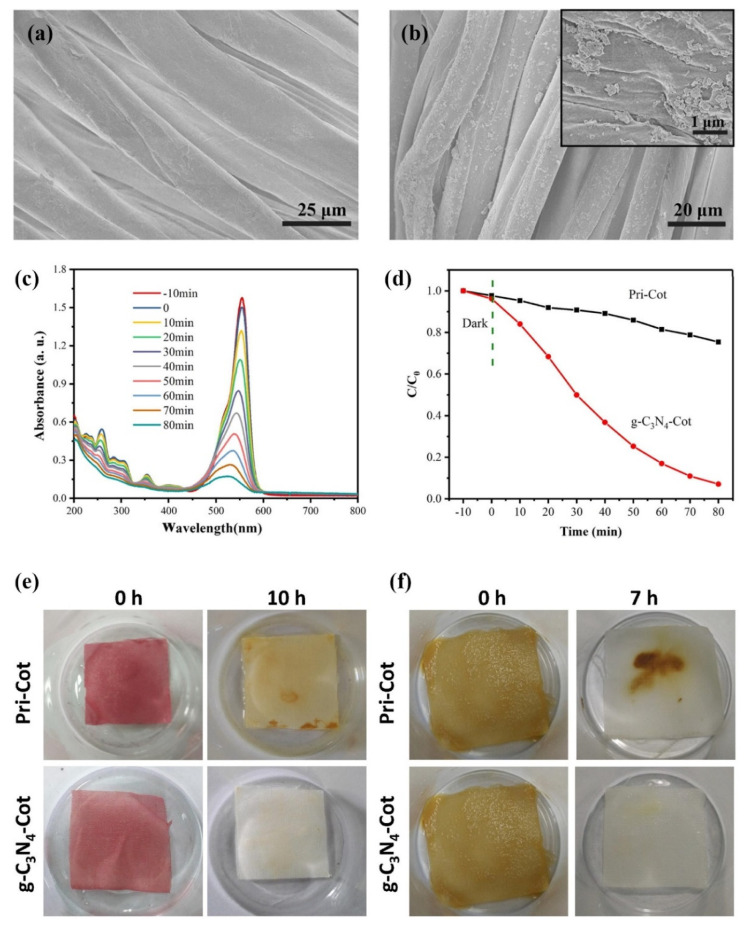
(**a**,**b**) SEM images of (**a**) untreated and (**b**) g-C_3_N_4_-coated cotton (insert—under higher magnification). (**c**) Evolution of UV–Vis absorption spectra of RhB solution with g-C_3_N_4_ coated cotton. (**d**) Plots of C/C_0_ of the RhB degradation versus time in the presence of uncoated pristine cotton (Pri-Cot) and g-C_3_N_4_ coated cotton (g-C_3_N_4_-Cot). (**e**–**f**) Degradation of white cotton samples stained with red wine (**e**) after 10 h of light irradiation under a xenon lamp and of white cotton samples stained with coffee (**f**) after 7 h of light irradiation under a xenon lamp. Reprinted with permission from [43]. Copyright 2018, Elsevier.

Among textile fibres, cotton has mostly been used for the application of g-C_3_N_4_ [40,41,42,43,44,45,46]. When g-C_3_N_4_ nanosheets were deposited onto the PDDA-modified cotton fabric (Figure 7) [43], the roughness of the cotton fibres increased, and the microscopic agglomerates of the g-C_3_N_4_ nanosheets were clearly visible on the SEM images (Figure 7a,b). 

The presence of the g-C_3_N_4_ coating induced the photocatalytic activity of the cotton sample under the irradiation of simulated light in the range of 350–780 nm, which enabled the photodegradation of the dye Rhodamine B (RhB) in an aqueous solution in contact with the coated sample. This resulted in a gradual decrease in RhB absorbance over time (Figure 7c). The photodegradation rate reached 90.2% after 80 min of irradiation, which was significantly higher than in the case of untreated cotton (Figure 7d). Moreover, the photocatalytic activity of the g-C_3_N_4_-coated cotton sample provided significant photocatalytic self-cleaning properties, enabling the discoloration of the red wine stain after 10 h of light irradiation and a coffee stain after 7 h of irradiation (Figure 7e,f). The electrostatic interactions between g-C_3_N_4_ nanosheets and PDDA provided high wash fastness of the coating with a degradation rate of 78.2%, even on the 13th reaction cycle.

**Figure 8:**
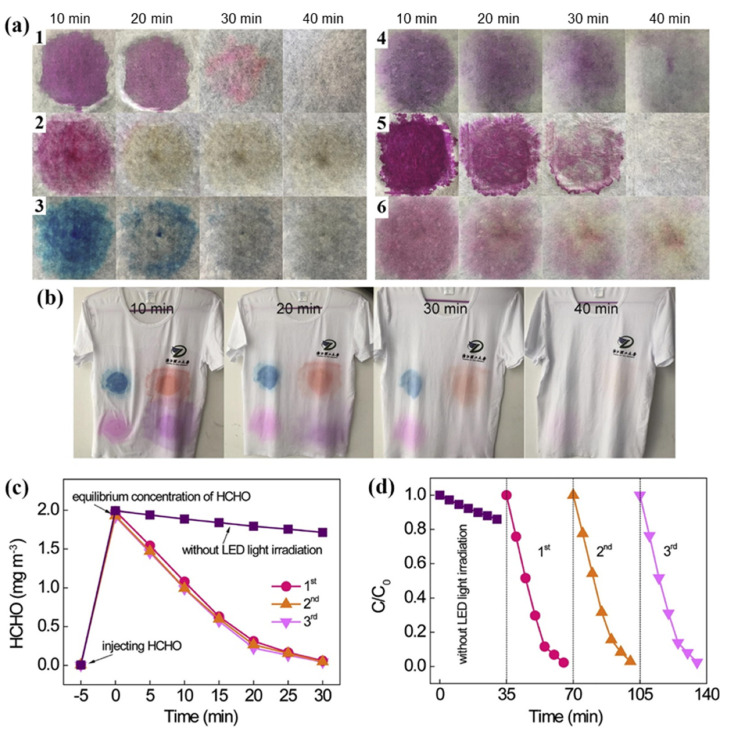
(**a**) The photocatalytic self-cleaning behaviour of g-C_3_N_4_ nanosheets modified textiles under sunlight illumination for 40 min: (1) RhB, (2) neutral red, (3) methyl blue, (4) reactive violet, (5) red pitaya juice, (6) waxberry juice; (**b**) the self-cleaning performance of a commercial T-shirt modified with g-C_3_N_4_ nanosheets; (**c**,**d**) the photocatalytic degradation of gaseous formaldehyde by a g-C_3_N_4_ nanosheet modified textile under irradiation with a LED lamp (50 W). Reprinted with permission from [44]. Copyright 2019, Elsevier.

Cotton with superior photocatalytic self-cleaning performance for removing stains of various coloured pollutants under solar light irradiation was also obtained by directly spraying a colloidal suspension of g-C_3_N_4_ nanosheets onto the fibre surface (Figure 8) [44]. 

A thin layer of g-C_3_N_4_ nanosheets was formed on the surface of the cellulose fibres through facilely spraying, which did not affect the hand feel and colour of the textile substrates. The abundant hydroxyl and amino groups of the g-C_3_N_4_ nanosheets formed by the alkaline exfoliation of the bulky material enabled the formation of hydrogen bonds between g-C_3_N_4_ and the cellulose fibres, which maintained the high stability of the modified textiles. The excellent photocatalytic self-cleaning properties of the textiles modified with the g-C_3_N_4_ nanosheets were confirmed by the rapid decolourization of various coloured pollutants such as RhB, neutral red, methylene blue, and reactive violet dye solutions at high concentrations as well as red pitaya juice and waxberry juice after 40 min under natural sunlight (Figure 8a). The blue-, red-, orange-, and purple-coloured stains were also rapidly discoloured on the modified commercial T-shirt (Figure 8b). In addition to their self-cleaning ability, the g-C_3_N_4_ nanosheet modified textiles showed photocatalytic performance in the removal of gaseous formaldehyde with 100% removal efficiency after 30 min under an LED lamp source (Figure 8c). 

**Figure 9:**
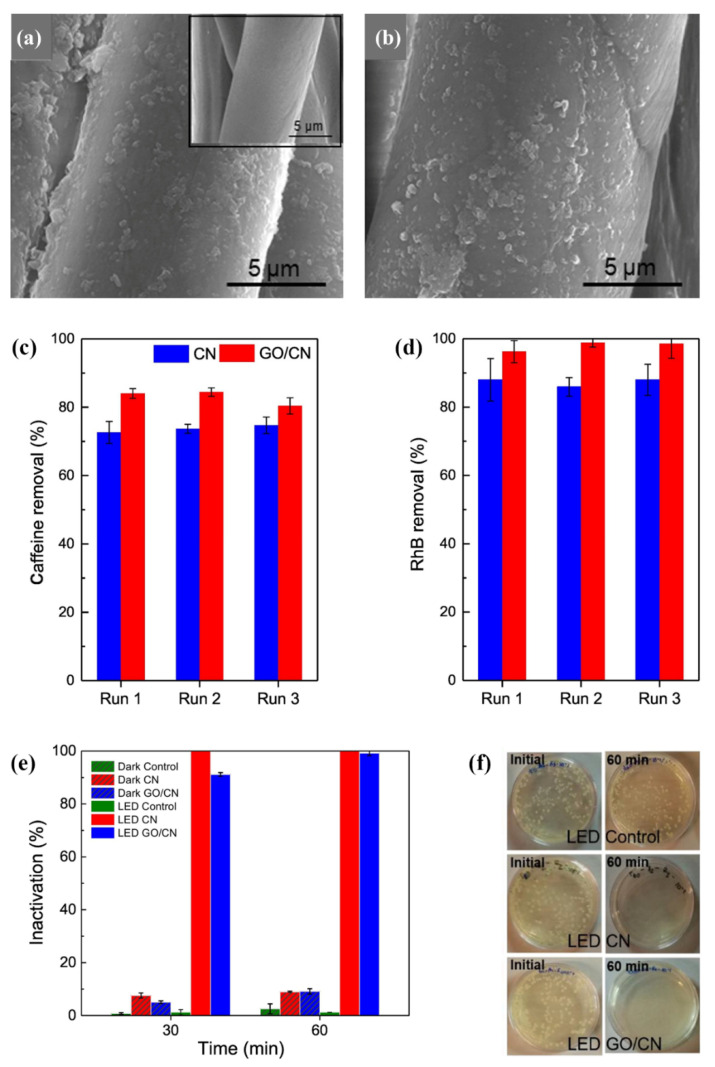
(**a**,**b**) SEM images of (**a**) g-C_3_N_4_ (an insert uncoated sample) and (**b**) GO/g-C_3_N_4_ coated cotton fabric samples. (**c**,**d**) Reusability assessment for the coated fabrics in the degradation of caffeine (**c**) and RhB (**d**) after a 240-min reaction. (**e**) *E. coli* inactivation with g-C_3_N_4_ and GO/g-C_3_N_4_ colloids and control experiments under visible light (LED) and dark conditions (dark). (**f**) Incubated agar plates inoculated with bacterial suspension and colloids or control before and after 60 min of LED irradiation. Reprinted with permission from [40]. Copyright 2019, Elsevier.

To increase the photocatalytic efficiency of g-C_3_N_4_, a small amount of GO suspension was added to the colloidal g-C_3_N_4_ and mixed for a certain period of time to produce the metal-free photocatalyst composite GO/g-C_3_N_4_ [40]. Both of the colloidal suspensions of GO/g-C_3_N_4_ and the single-component g-C_3_N_4_ were applied to cotton fibres by immersion at 40 °C for 3 h, squeezing, and thermofixation at 100 °C for 4 min. According to the SEM images (Figure 9a,b), micro- and sub-micro agglomerates of the g-C_3_N_4_ nanosheets and GO were relatively uniformly distributed on the cotton fibre surface. The morphology of the fibre surface remained unchanged after three reuse cycles, indicating their good stability under the applied experimental conditions. The results of the photocatalytic self-cleaning activity showed that the presence of GO in the heterojunction with g-C_3_N_4_ increased the self-cleaning performance compared to a single-component g-C_3_N_4_ coating and enabled an almost complete degradation of caffeine and RhB after 360 min (Figure 9c,d). The enhanced photocatalytic activity of GO/g-C_3_N_4_ against the tested pollutants was attributed to GO, which, due to the increased surface area, can act as a co-adsorbent for organic pollutants. Moreover, upon photoexcitation, electrons are transferred from g-C_3_N_4_ to GO, the latter acting as an electron scavenger to stabilize the recombination of the photogenerated electron–hole pairs. Both the GO/g-C_3_N_4_ and g-C_3_N_4_ colloids caused the inactivation of the Gram-negative bacterium *Escherichia coli*, which was higher than 99.2% after 60 min of contact under visible light irradiation (Figure 9e,f). As expected, the colloids did not show photocatalytic antibacterial activity under dark conditions.

Another promising alternative for the degradation of liquid and gaseous environmental pollutants is the two-semiconductor TiO_2_/g-C_3_N_4_ composite coating prepared by the LbL self-assembly strategy for the deposition of TiO_2_ nanoparticles and g-C_3_N_4_ nanosheets on the cotton fabric to generate two, five, or seven bilayers [42]. The combination of TiO_2_ with g-C_3_N_4_ in the coating extended the range of the light response and enhanced the photocatalytic activity under visible light; this phenomenon increased with an increase in the number of bilayers. Accordingly, seven bilayers led to a maximum degradation rate of 92.5% for RhB, but all bilayers degraded more than 90% of the toluene within 50 min under simulated sunlight irradiation.

**Figure 10:**
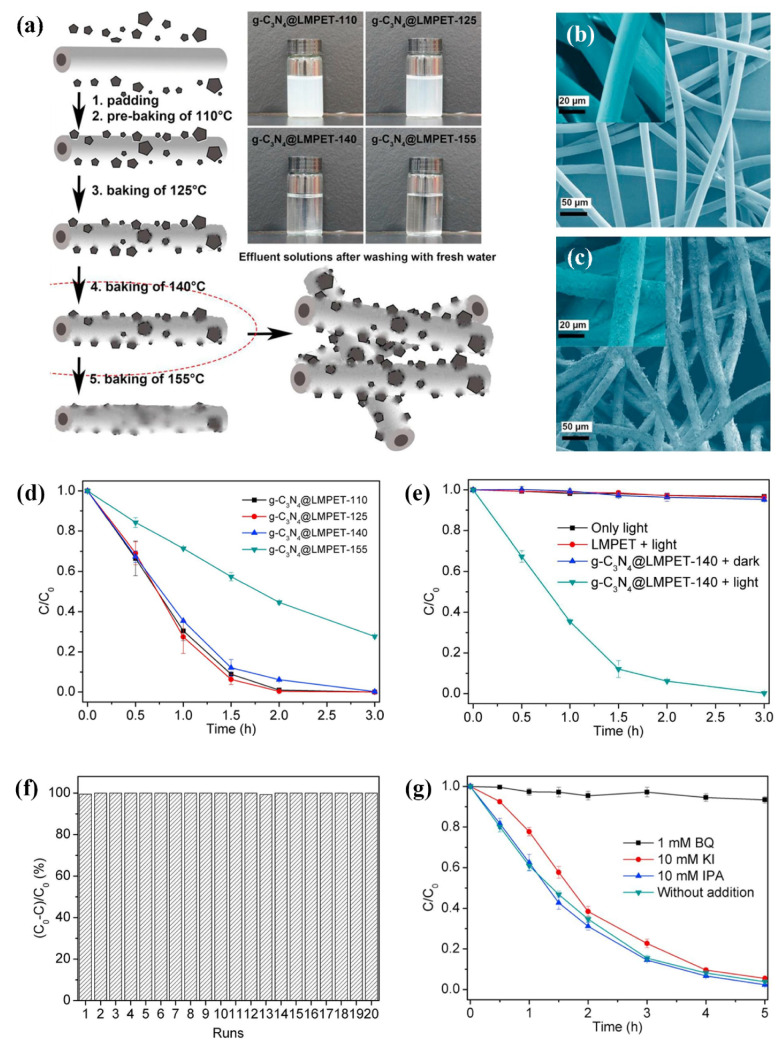
(**a**) Schematic presentation of the effect of curing (baking) temperature on fibre morphology and the corresponding effluent solutions after washing with fresh water. SEM images of (**b**) untreated LMPET sample and (**c**) g-C_3_N_4_@LMPET-140 sample cured at 140 °C (b2). (**d**) Photocatalytic degradation of sulfadiazine (SDZ) by g-C_3_N_4_@LMPET-X samples cured at different temperatures (X corresponds to the curing temperature) under solar irradiation. (**e**) SDZ degradation by g-C_3_N_4_@LMPET-140 sample in different conditions. (**f**) Cyclic photocatalytic degradation of SDZ by g-C_3_N_4_@LMPET-140 under solar irradiation for 3 h. (**g**) Effect of trapping agent on the photocatalytic degradation of SDZ under solar irradiation. Reprinted with permission from [51]. Copyright 2018, Elsevier.

To provide a medium for the detoxification of nerve agents, a nanocomposite of copper nitrate hemipentahydrate, 1,3,5 benzenetricarboxylic acid, and oxidized g-C_3_N_4_ was synthesized and applied to the cotton fabric [41]. Incorporation of the oxidized g-C_3_N_4_ into Cu-BTC not only resulted in mesoporosity but also drastically increased the surface reactivity. As a result, the detoxification of the nerve gas surrogate dimethyl chlorophosphite (DMCP) dramatically increased. The adsorption and detoxification processes were accompanied by a visible and gradual colour change, whereby the modified textiles totally lost their turquoise colour and became yellowish after 90 min of the adsorption of DMCP vapours. Supreme adsorption of the chemical warfare agent and its decomposition to nontoxic compounds was indicated after 192 h of exposure to ambient light. Therefore, this material can be referred to as a “smart textile” because of its ability to simultaneously adsorb, degrade, and detect the vapours or droplets of the toxic surrogate.

g-C_3_N_4_ has also been used to modify the surface of polyester fibres [51] as well as polyester/viscose blends [54] to construct a photocatalytic textile platform. In both cases, to overcome the problem of bridging g-C_3_N_4_ to polyester fibres, a hot-melt adhesive method was used to embed g-C_3_N_4_ on the surface of a low-melting-point sheath-core composite polyester (LMPET). A nonwoven fabric of g-C_3_N_4_@LMPET was prepared by applying a g-C_3_N_4_ suspension onto the LMPET fabric samples by dip-padding, squeezing, and pre-curing (pre-baking) at 110 °C for 30 min. The preheated samples were then cured (baked) at four temperatures: 110 °C, 125 °C, 140 °C, and 155 °C, for 30 min (Figure 10a–c). It was found that the selection of the correct curing temperature is of great importance, as this temperature directly affects the rate of g-C_3_N_4_ immobilisation as well as the photocatalytic activity. Namely, the washing test after curing showed that the effluent solutions of the g-C_3_N_4_@LMPET samples cured at 110 °C and 125 °C were turbid and contained high levels of g-C_3_N_4_, while the solutions of the g-C_3_N_4_@LMPET samples cured at 140 °C and 155 °C were quite clear, indicating that only the latter two temperatures could achieve the solid immobilisation of g-C_3_N_4_ (Figure 10b,c). As determined by the degradation of the antibiotic sulfadiazine, the photocatalytic activity of the g-C_3_N_4_@LMPET sample cured at 155 °C decreased significantly compared to the samples cured at lower temperatures (Figure 10d). The reason for this could be the coating of the g-C_3_N_4_ particles by the LMPET, which resulted in a reduction of the exposed part of the catalyst. Therefore, a curing temperature of 140 °C was found to be the most suitable (sample code: g-C_3_N_4_@LMPET-140). The photocatalytic activity was also directly affected by the dark and light conditions. While the g-C_3_N_4_@LMPET-140 sample showed no photodegradation performance against sulfadiazine (SDZ) under dark conditions and the concentration of SDZ remained practically unchanged, 99.8% of the SDZ was degraded by g-C_3_N_4_@LMPET-140 under simulated sunlight irradiation for 3 h (Figure 10e). Moreover, the degradation effect after 3 h was not diminished even after repeating the experiment 20 times, indicating good binding fastness between g-C_3_N_4_ and LMPET and multiple uses of this catalytic platform (Figure 10f). In investigating the mechanism of the photodegradation of SDZ by g-C_3_N_4_, three trapping agents were used, i.e., p-benzoquinone (BQ) as a O_2_^∙^^−^ scavenger, potassium iodide (KI) as a hole scavenger, and isopropanol (IPA) as a ^∙^OH scavenger (Figure 10g). Since the degradation rate of SDZ did not change appreciably in the presence of KI and IPA, BQ reduced the overall degradation rate of the SDZ to zero, indicating that O_2_^∙^^−^ is the major active ROS species in the photocatalytic degradation system of SDZ.

**Figure 11:**
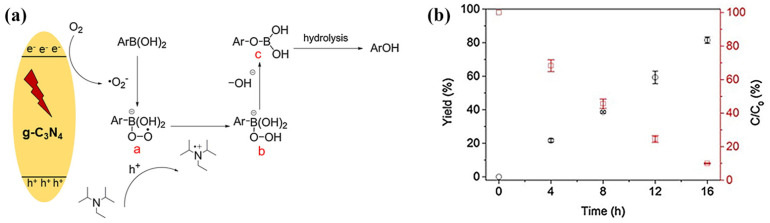
(**a**) Schematic diagrams of possible reaction mechanisms over g-C_3_N_4_ under solar light irradiation. (**b**) Time courses of oxidative hydroxylation of arylboronic acid using g-C_3_N_4_/LMPET under solar light irradiation (yield—black; residual rate C/C_0_—red). Reprinted with permission from [52]. Copyright 2019, Elsevier.

The same g-C_3_N_4_-modified LMPET fabric was also used for the first time as a photocatalytic platform for the oxidative hydrolysis of arylboronic acids to phenols, with isopropanol as a solvent in the presence of N,N-diisopropylethylamine under visible light irradiation and in the absence of transition-metal catalysts (Figure 11a) [52]. In this reaction, a high yield of phenols, 82%, was obtained after 16 h at ambient pressure and room temperature, using air as the terminal oxidant source (Figure 11b). The g-C_3_N_4_/LMPET fabric also showed excellent conversion efficiency after eight cycles. This opened up the possibility of using a composite textile material for photocatalytic organic transformations.

**Figure 12:**
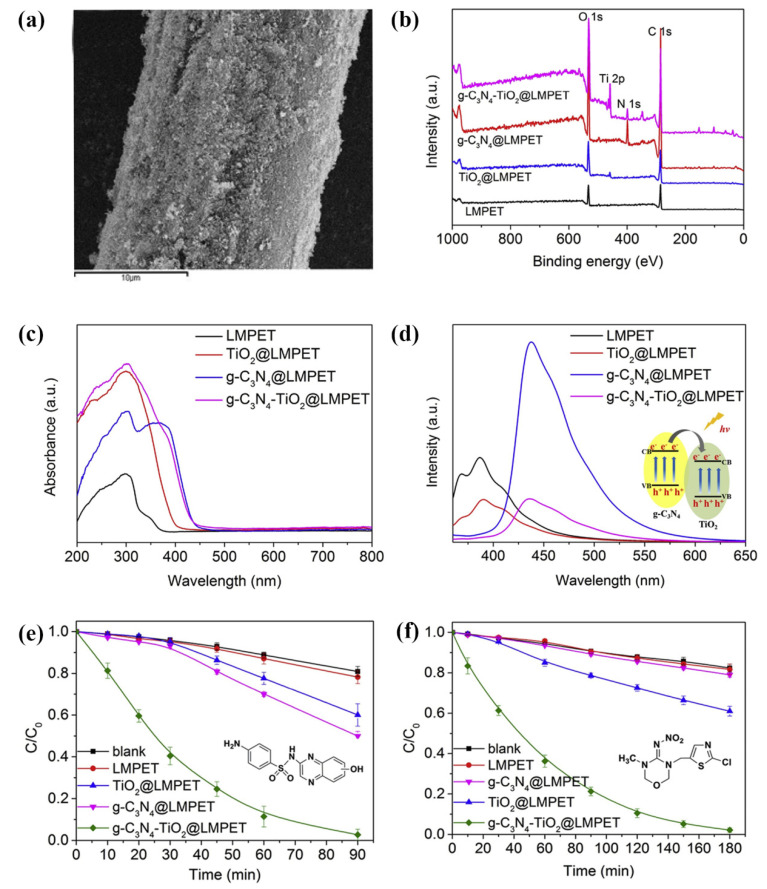
(**a**) Morphology of the g-C_3_N_4_-TiO_2_@LMPET surface. (**b**) XPS survey spectra. (**c**) UV–Vis diffuse reflectance absorption spectra of LMPET, TiO_2_@LMPET, g-C_3_N_4_@LMPET, and g-C_3_N_4_-TiO_2_@LMPET. (**d**) Photoluminescence (PL) spectra of LMPET, TiO_2_@LMPET, g-C_3_N_4_@LMPET, and g-C_3_N_4_-TiO_2_@LMPET. (**e**,**f**) Photocatalytic degradation of sulfaquinoxaline (SQX) (**e**) and thiamethoxam (**f**) under different conditions exposed to solar irradiation (SQX = 2 × 10^−5^ mol/L, thiamethoxam = 2 × 10^−5^ mol/L, pH 7). Reprinted with permission from [53]. Copyright 2019, Elsevier.

**Figure 13:**
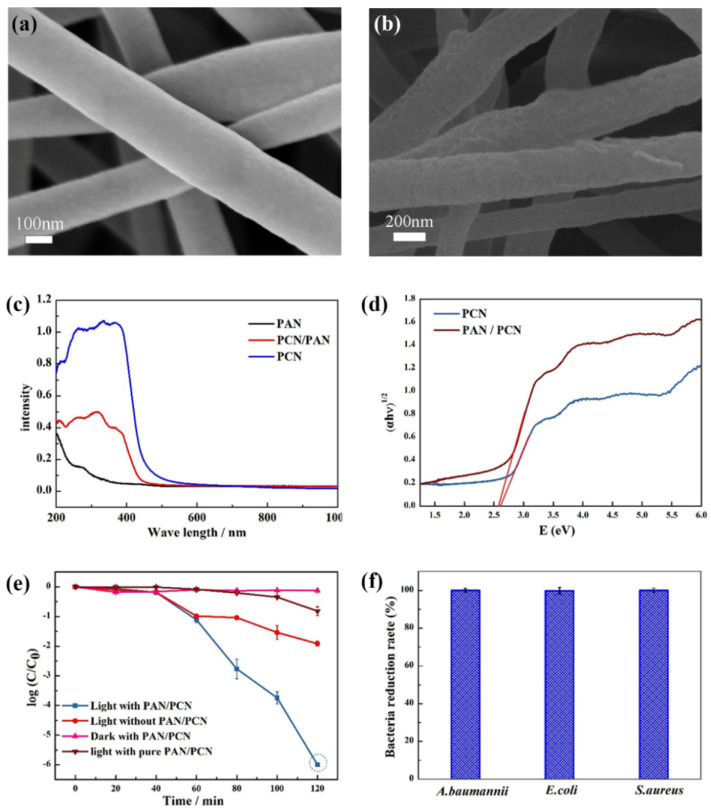
(**a**,**b**) SEM images of pure PAN (**a**) and PAN/g-C_3_N_4_ composite (**b**) fabrics. (**c**) UV–Vis diffuse reflectance images of PAN fabric, PAN/g-C_3_N_4_ (PAN/PCN) composite fabric, and g-C_3_N_4_ (PCN). (**d**) Plots of converted Kubelka–Munk functions versus light energy of g-C_3_N_4_ (PCN) and PAN/g-C_3_N_4_ (PAN/PCN) composite fabric. (**e**) Photocatalytic disinfection of *Staphylococcus aureus*. (**f**) Percentage of disinfection efficiency of *Acinetobacter baumannii*, *Escherichia coli*, and *Staphylococcus aureus*. Reprinted with permission from [47]. Copyright 2020, Elsevier.

To solve the problem of the photoetching of the polyester fibre surface as an organic carrier of photocatalyst, the fibre surface was coated with two-dimensional g-C_3_N_4_ sheets prior to the deposition of TiO_2_ [53]. In the thus-prepared g-C_3_N_4_-TiO_2_ composite coating on the PET fibre surface, the g-C_3_N_4_ layer acted as a barrier between the fibre and TiO_2_ particles to protect the PET from the ^∙^OH etching. Simultaneously, the photocatalytic performance of TiO_2_ under solar irradiation was enhanced [53]. In the experiment, a uniform suspension of g-C_3_N_4_ was first deposited on the LMPET by the pad–dry–cure method with a curing temperature of 135 °C to melt the LMPET sheath to adhere to g-C_3_N_4_. The obtained g-C_3_N_4_@LMPET sample was then immersed in the prepared TiO_2_ suspension, and the deposition of TiO_2_ on the g-C_3_N_4_@LMPET sample was achieved by a one-step hydrothermal reaction, which was conducted at 120 °C for 2 h. g-C_3_N_4_@LMPET and TiO_2_@LMPET samples were also prepared for comparison. The surface attachment of the g-C_3_N_4_ and TiO_2_ particles to the LMPET caused the g-C_3_N_4_-TiO_2_@LMPET sample to become blurrier and rougher (Figure 12a). The major elements present on the sample surface, including C, O, N, and Ti, were detected through XPS analysis (Figure 12b). The UV–VIS absorption spectra (Figure 12c) revealed the absorption band in the UV region for LMPET due to the benzene ring structure. The intensity of this band increased for the TiO_2_@LMPET sample due to the efficient UV light harvesting by TiO_2_. In the g-C_3_N_4_@LMPET sample, a red shift and the broadening of this absorption band occurred due to the characteristic absorption of g-C_3_N_4_ in the range of 200–450 nm. Consequently, the absorption band of the g-C_3_N_4_-TiO_2_@LMPET sample showed a red shift of the absorption edge and an increased absorption intensity compared to the TiO_2_@LMPET sample, indicating the synergistic effect of TiO_2_ and g-C_3_N_4_ in the composite. The formation of the TiO_2_/g-C_3_N_4_ heterojunction was also confirmed by the photoluminescence spectra (Figure 12d), in which the intensity of the emission peak around 450 nm of the g-C_3_N_4_-TiO_2_@LMPET sample was much lower than that of the g-C_3_N_4_@LMPET sample. This suggested that the electrons in the CB could be transferred from g-C_3_N_4_ to TiO_2_ and that they efficiently suppressed the recombination of the photogenerated electron–hole pairs. The extended light absorption and electron transfer between g-C_3_N_4_ and TiO_2_ as well as a larger number of active sites led to a significant improvement in the photocatalytic performance of the g-C_3_N^4^-TiO_2_@LMPET sample in the degradation of the antibiotic sulfaquinoxaline (Figure 12e) and the pesticide thiamethoxam (Figure 12f) under solar irradiation compared to the TiO_2_@LMPET and g-C_3_N_4_@LMPET samples. Thus, a removal rate of more than 97% was achieved within 90 min for sulfaquinoxaline and within 180 min for thiamethoxam. In contrast, the removal rate of the TiO_2_@LMPET and g-C_3_N_4_@LMPET samples did not exceed 50% for either contaminant. The g-C_3_N_4_-TiO_2_@LMPET showed good repeatability in cyclic experiments, with a removal rate of 97%, even after 10 reaction cycles, indicating high catalytic activity and the reusability of the g-C_3_N_4_-TiO_2_@LMPET sample.

g-C_3_N_4_ nanosheets were also incorporated into the bulk of synthetic fibres, where polyacrylonitrile [47,48,49,50] and polyethylene terephthalate [55,56] as well as polylactic acid [57] were used as polymer matrices for the incorporation of g-C_3_N_4_. In these cases, g-CN was dispersed in the polymer solution and was used as a spinning solution for the production of the nonwoven fabrics. For example, in the case of polyacrylonitrile (PAN) [47], the ultrasonically prepared uniform dispersion of g-C_3_N_4_ in N,N-dimethylformamide was mixed with PAN and used as an electrospinning solution to prepare the nonwoven composite fabric. The incorporation of g-C_3_N_4_ transformed the smooth surface of PAN into a rough and corrugated surface, indicating the tight incorporation of the g-C_3_N_4_ nanosheets into the PAN structure (Figure 13a,b). Moreover, the presence of g-C_3_N_4_ nanosheets caused the light absorption of the PAN/g-C_3_N_4_ composite fabric to be red-shifted to the visible light region with a band gap energy of 2.55 eV, which was very similar to the band gap energy of the g-C_3_N_4_ nanosheets of 2.58 eV (Figure 13c,d). The composite fabric showed excellent photocatalytic disinfection performance with the inactivation of *Staphylococcus aureus*, *Acinetobacter baumannii,* and *Escherichia coli* within 120 min (Figure 13e,f). The excellent disinfection performance of this composite fabric is caused by the killing of the tested bacteria by the synergistic effect of photogenerated holes (h^+^) and H_2_O_2_ rather than by the inhibitory and adhesion bacteria effect. Due to the high filtration capacity, excellent stability, and long-term durability, the as-prepared composite fabric is very promising for water filtration and disinfection.

**Figure 14:**
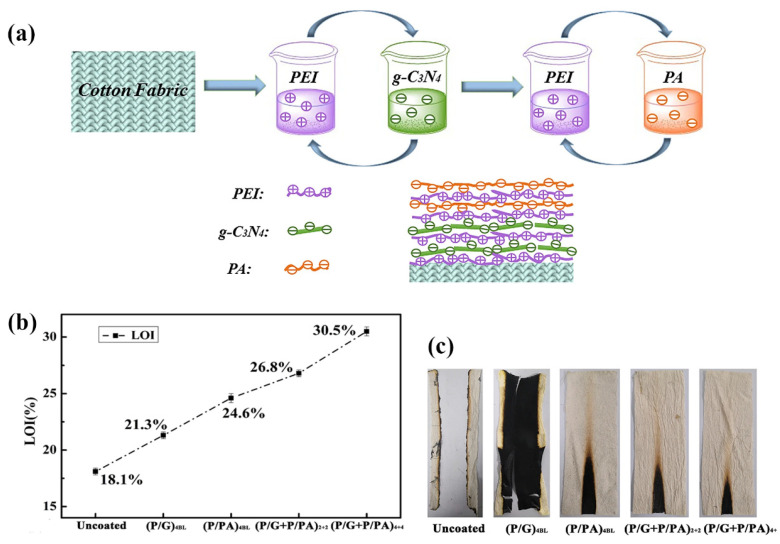
(**a**) Diagrammatic illustration of layer-by-layer (LbL) assembly of cotton fabric. (**b**) LOI values of uncoated and different treated samples. (**c**) Images following the vertical flame test of uncoated and different treated samples. Reprinted with permission from [46]. Copyright 2021, Springer Nature.

In addition to photocatalytic performance, the effect of the presence of g-C_3_N_4_ in the coating on the thermal stability and the flame retardancy of cotton fibres was also investigated [46]. Namely, it was assumed that g-C_3_N_4_ was a two-dimensional layered polymeric structure, which when in reach of a nitrogen element, can form a thin insulating membrane for a flame-retardant coating. In this case, the layer-by-layer (LbL) self-assembly technique based on the electrostatic attraction between oppositely charged polyelectrolytes or nanoparticles was used to prepare two or four insulating bilayers (BLs) by alternating immersion in the cationic polyethyleneimine (PEI) solution and the anionic suspension of g-C_3_N_4_ followed by the preparation of two or four intumescent BLs by alternating immersion in the PEI and the anionic phytic acid (PA) solutions (Figure 14a). A total of four PEI/g-C_3_N_4_ BLs and four PEI/PA BLs were also prepared for comparison. It was found that the presence of four PEI/g-C_3_N_4_ insulating bilayers initiated the start of thermal thermo-oxidative decompositions of the coated cotton samples at lower temperatures compared to the untreated cotton, which was followed by a significant reduction of the weight loss rate and a significant increase in the char residue at 700 °C. These results imply that the coating inhibited the thermal decomposition process of the underlying cotton fibre. The presence of four PEI/g-C_3_N_4_ bilayers also increased the value of the limiting oxygen index from 18.1% for the untreated cotton to 21.3% (Figure 14b) and left an integrated char after burning (Figure 14c). In the case of the combined PEI/g-C_3_N_4_ and PEI/PA coatings, the synergistic effect between the insulating action of g-C_3_N_4_ and the intumescent action of PA resulted in a significant increase in the flame-retardant properties and exhibited the phenomenon of self-extinguishing regardless of the number of BLs (Figure 14c). It is believed that the g-C_3_N_4_ layer in the coating can act as a condensed phase barrier, which, when formed under an intumescent coating, can act as a support for the latter by enhancing the thickness and the strength of the intumescent layer, thus providing effective protection for the underlying cotton fibres during combustion.

## 4. Conclusions and Future Perspectives

The development of non-metallic and environmentally friendly photocatalysts is of high importance for controlling environmental pollution and for coping with the energy crisis. The visible-light-driven metal-free semiconductor photocatalyst g-C_3_N_4_ has attracted increasing attention due to its nontoxicity and easy conversion of solar energy to chemical energy. The reviewed literature reporting on the application of g-C_3_N_4_-based photocatalysts to flexible and sustainable textile materials serving as photocatalyst carriers indicates that textiles represent attractive potential alternatives to inorganic material-based carriers. The textile materials carrying g-C_3_N_4_-based photocatalysts on the surface or in the bulk have great potential for practical applications, providing high durability of these photocatalytic systems as well as their reusability in catalysis reactions involved in the environmental remediation and selective organic transformation processes. 

The other possible application of textile materials carrying g-C_3_N_4_-based photocatalysts on the surface or in the bulk is in the field of multifunctional textile materials with self-cleaning properties, antibacterial properties, thermal stability, and flame retardancy. The self-cleaning properties of these textiles has the potential to reduce washing frequency, whereas the antibacterial properties could provide protection from potentially pathogenic microorganisms. Additionally, g-C_3_N_4_ is also an important flame retardant, as its structural characteristics provide a nitrogen rich flame retardant with the great ability to induce the formation of protective char barrier layers protecting the underlying polymeric materials.

This review summarizes the results achieved in the applications of g-C_3_N_4_-based photocatalysts with textile materials and proves that the research community has only scratched the surface in this field. Future research focusing on the production of stable nanosized g-C_3_N_4_ particles able to preserve the nanometric scale and photocatalytic efficiency after application to a textile as well as on the design of applications for different g-C_3_N_4_-based photocatalysts could be an important step towards lowering the effective concentration of the applied photocatalysts while preserving their high durability and reusability. Studying the interactions of the textile fibre surfaces with g-C_3_N_4_-based photocatalysts as well as with generated photocatalytic radical species would enable us to temper the destructive nature of the latter towards fibres. Finally, recycling strategies should be considered as the basis for the further development of novel, effective, and sustainable textile materials functionalized with g-C_3_N_4_-based photocatalysts.

## Figures and Tables

**Table 1:** Textile fibres, g-C_3_N_4_ source, and its modifications, application methods, and developed functionalities.

Fibres	g-C_3_N_4_ Precursor	Modification of g-C_3_N_4_	Application Method	Developed Functionality	Ref.
Cotton	Dicyandiamide	GO	Immersion (40 °C, 3 h), squeezing, drying (100 °C, 4 min)	Photocatalytic degradation, antibacterial properties	[40]
Cotton	Dicyandiamide	Cu oxides/hydroxides— 1,3,5-benzene tricarboxylate	Alternate immersion (25 °C, 5 min), heating (75 °C); drying (100 °C, 16 h)	Sensing, photocatalytic detoxification	[41]
Cotton	Melamine	TiO_2_	Layer-by-layer self-assembly	Photocatalytic degradation	[42]
Cotton	Melamine	Unmodified	Immersion (80 °C, 2 h), rinsing, drying	Photocatalytic degradation, self-cleaning	[43]
Cotton	Melamine	Unmodified	Spraying, drying (60 °C, 12 h)	Photocatalytic degradation, self-cleaning	[44]
Cotton	Urea	TiO_2_	Immersion (10 min), squeezing, heating (130 °C and 60 °C, 30 min)	Photocatalytic degradation, antibacterial properties	[45]
Cotton	Urea	Phytic acid	Layer-by-layer self-assembly	Thermal stability, flame retardancy	[46]
Polyacrylonitrile	Urea	Unmodified	Electrospinning	Photocatalytic disinfection, antibacterial properties	[47]
Polyacrylonitrile	Urea	Unmodified	Electrospinning	Photocatalytic degradation	[48,49,50]
Polyester	Urea	Unmodified	Dip-padding, prebaking (110 °C), baking (110 °C, 125 °C, 140 °C, 155 °C, 30 min)	Photocatalytic degradation, photocatalytic organic transformation	[51,52]
Polyester	Urea	TiO_2_	Pad-dry-cure for g-C_3_N_4_; in situ hydrothermal (120 °C, 2 h) for TiO_2_	Photocatalytic degradation	[53]
Polyester/ Viscose	Urea	Unmodified	Double-dip-double-nip, prebaking (110 °C), baking (140 °C, 30 min)	Photocatalytic degradation	[54]
Polyethylene terephthalate	Urea	Unmodified	Electrospinning	Photocatalytic degradation	[55,56]
Polylactic acid	Urea	Unmodified	Electrospinning	Photocatalytic degradation	[57]

## Data Availability

The data presented in this study are available on request from the corresponding author.
